# The Impact of the Parental Patterns of Morbidity and Comorbidity in the Cross-Generational Transmission of Risk for Major Depression and Alcohol Use Disorder

**DOI:** 10.1002/ajmg.b.33052

**Published:** 2025-08-14

**Authors:** Kenneth S. Kendler, Linda Abrahamsson, Jan Sundquist, Kristina Sundquist

**Affiliations:** 1 Virginia Institute for Psychiatric and Behavioral Genetics, Virginia Commonwealth University, Richmond, Virginia, USA; 2 Department of Psychiatry, Virginia Commonwealth University, Richmond, Virginia, USA; 3 Center for Primary Health Care Research, Lund University, Malmö, Sweden

**Keywords:** alcohol use disorder, dual-mating study, major depression, parent-offspring transmission

## Abstract

To further understand the inter-relationship of the familial transmission of major depression (MD) and alcohol use disorder (AUD), we examine, via a multivariable Cox proportional hazards model, risks for AUD and MD in 1,244,516 individuals born in Sweden from 1970 to 1990 to intact mother–father pairs as a function of parental diagnoses of MD and/or AUD. Across the nine possible mating types, we see both direct transmission (MD → MD, AUD → AUD) and also, less strongly, indirect transmission: MD → AUD and AUD → MD. Risks in offspring accumulate with multiple affected parents, which reveals the impact of interactive effects in risk prediction. Interestingly, the risk for comorbid AUD/MD in offspring is higher when one parent has MD and the other AUD rather than when one parent has both disorders. Modest sex effects are seen, with maternal-offspring transmission sometimes significantly stronger than paternal-offspring transmission. In most comparisons, parental-offspring transmission was modestly stronger for same-sex versus opposite-sex parent-offspring pairs. These results suggest that MD/AUD comorbidity in Sweden is due, at least in part, to correlated familial liability transmitted by direct and indirect paths across generations. We could reject the hypothesis that an AUD/MD syndrome was specifically transmitted from parents to offspring.

## 1 ∣ Introduction

Comorbidity is a central feature of psychiatric illness seen both in the clinical and in population samples (Kessler 1997; Kessler et al. 1993; Maser and Cloninger 1990; Oldham et al. 1995). Furthermore, comorbidity has long been known to impact patterns of familial aggregation (Biederman et al. 1992; Merikangas et al. 1994; Pickens et al. 1995). However, with some exceptions, in the large majority of psychiatric genetic investigations—be it family, twin, or molecular genetic studies—disorders are treated without consideration for co-occurring conditions. A range of conceptual approaches have been taken to the analysis of comorbidity, but most of them are “top-down,” beginning with statistical models from which statistical predictions are made that can then be evaluated empirically (Klein and Riso 1993; Neale and Kendler 1995; Rhee et al. 2002).

In this paper, we take the opposite approach by revisiting an older methodology in psychiatric genetics—the Dual-Mating Study (Gottesman and Bertelsen 1989; Kendler and Klee 2024; Luxenburger 1939). As outlined by Gottesman and Bertelsen (1989), the original dual mating design was, as they termed it, “homotypic”—where both parents were affected with the same disorder—a strategy used for both schizophrenia and manic-depressive illness. But they advocated for a “heterotypic” dual mating design in which patterns of illness were systematically explored when the two parents had different psychiatric illnesses. They implemented this approach using the Danish Psychiatric Register (Gottesman and Bertelsen 1989).

We here adopt a heterotypic dual mating strategy, utilizing the resources of the Swedish national registers, to understand the familial underpinnings of the comorbidity between two important and relatively commonly comorbid disorders, major depression (MD) and alcohol use disorder (AUD) (Brière et al. 2014; Grant and Harford 1995; Merikangas and Gelernter 1990; Swendsen et al. 1998), whose transmission within families has been examined previously (Kendler et al. 1993, 2022; Maier et al. 1994; Merikangas et al. 1994; Nurnberger Jr. et al. 2004; Prescott et al. 2000; Winokur 1979). In particular, in prior Swedish analyses, using family genetic risk scores, we found that the genetic risk for MD and AUD interacted on an additive scale with each other in predicting risk for both MD and AUD (Kendler et al. 2022). Estimated heritability and shared environmental effects for the two disorders are for MD 37% and 0% (Sullivan et al. 2000) and for AUD 49% and 10% (Verhulst et al. 2015). Genetic correlations between MD and AUD have been estimated to equal +0.39 in twin studies (Kendler et al. 1993) and +0.53 using polygenic risk scores (Foo et al. 2018).

Because of the large sample sizes available using Swedish national registries, we have in these analyses the capacity to disentangle potentially subtle features of the parent-offspring transmission of these two disorders.

The questions we seek to address include the following:

1. What are the magnitudes of parent-offspring transmission for MD and for AUD?
2. Do the magnitudes of these transmissions differ from fathers versus mothers?
3. Does the impact of parental MD on risk for offspring MD or parental AUD on offspring AUD differ if the other parent does versus does not have the same disorder?
4. Does parental transmission of MD and AUD to sons and to daughters differ?
5. Does the specificity of transmission differ for MD versus AUD?
6. Is MD-AUD comorbidity specifically transmitted across generations?

## 2 ∣ Methods

Nationwide Swedish registers, along with a nearly complete nationwide primary health care research database, were used for finding information on study individuals (Table A1). The unique identification numbers of each individual, replaced with a serial number for confidentiality, were used for linkages between data sources. Ethical approval was secured from the regional ethical review board in Lund, Sweden.

We identified all individuals born in Sweden 1970–1990, with available follow-up to at least age 15 (i.e., no deaths or emigrations) *n* = 2,071,848. These individuals, here called offspring, had to be raised in an intact family, defined as living in the same household as the biological mother and the biological father from birth to age 15, leading to a sample size of *n* = 1,244,516. The multigeneration register was used to find biological parents. Every fifth year, between the years 1970 to 1985, household identification numbers were updated in the Population and Housing Census. For years with no information on whether offspring and parents were registered at the same residence, we made use of data from the closest year. Every year from 1986 onward, we used the family identification from the Total Population Register, defined as individuals registered at the same property who are related, married, or have common children.

Diagnoses of AUD and MD in offspring and parents were searched for using the Swedish Hospital Discharge Register, Outpatient Care Register, primary care data, the Swedish Prescribed Drug Register, the Swedish Cause of Death Register, the Swedish Criminal Register, and the Swedish Suspicion Register (for details, see Table A2).

In intact families, we studied offspring from the age of 10, using a multivariable Cox proportional hazards model with the age of offspring as the time scale, the effect of lifetime parental diagnoses of AUD and MD on time to first diagnosis of MD or AUD as three separate outcomes: (i) AUD total, (ii) MD total, and (iii) comorbid MD-AUD. The offspring were followed until diagnosis, end of study (December 31, 2018), death, or emigration—whichever occurred first. We utilized two different types of model set-ups for the parental diagnostic combinations. In all models, we control for sex and year of birth of offspring and year of birth of the mother and father. To remove problems with nonproportional hazards, we include interaction effects between the age of offspring and these controlling variables by using a procedure of splitting data over time (Zhang et al. 2018). We calculate contrasts for testing whether the effects of different parental dual diagnostic combinations were equal to each other.

We define a parent without any AUD or MD diagnoses as a “null” parent, and let the null × null dual parental diagnostic combination be the reference level (labeled A) to which we compare the other nine possible mating combinations, labeled B–J, in order of frequency of combination seen in data. In the first model set up, we include independent dummy variables for all combinations, B: AUD^+^MD^−^ × null, C: AUD^−^MD^+^ × null, D: AUD^+^MD^+^ × null, E: AUD^−^MD^+^ × AUD^−^MD^+^, F: AUD^−^MD^+^ × AUD^+^MD^−^, G: AUD^+^MD^+^ × AUD^−^MD^+^, H: AUD^+^MD^−^ × AUD^+^MD^−^, I: AUD^+^MD^+^ × AUD^+^MD^−^, and J: AUD^+^MD^+^ × AUD^+^MD^+^. In the second setup, to estimate sex-specific parental effects, we remove the independent variables of dual parental diagnostic combinations to instead include four independent variables of Mother's AUD, Mother's MD, Father's AUD, and Father's MD, along with all possible two-, three-, and four-way interactions. To remove problems with over-fitting of the model, the final model variables were chosen from applying a backwards selection procedure based on likelihood ratio testing and a significance level of 0.05, removing one variable at a time. As an extension to the second model type, to further analyze the effect of sex-specific parental AUD and MD on sons and on daughters, we included in the model interaction effects with sex of offspring and the mother and father AUD and MD variables. The final set of covariates were chosen with the same backwards selection procedure, with the additional criteria that a parental diagnostic variable was included even if not significant, as long as the offspring sex interaction with that variable was significant.

For testing whether transmission of risk for AUD (non-comorbid with MD) is more specific in comparison to MD (non-comorbid with AUD), in a case–case study design (Pogreba-Brown et al. 2020), we include all offspring cases with AUD only (*n* = 18,345) or MD only (*n* = 174,011), in a first model defining the AUD only individuals as the cases of interest and the MD only individuals as the comparison cases, and in a second model running the opposite model. Firstly, logistic regression with AUD case status as the dependent variable is performed, including all different dual mating combinations as independent variables, with the UN × UN combination as the reference level, and further controlled for birth years of offspring and parents, as well as sex of offspring. Secondly, the same model is run with MD case status as the dependent variable. We define F as the fraction of interest, being the OR for exposure to one parent with AUD, for AUD cases vs. MD cases, measured from the first model, divided by the OR for exposure to one parent with MD, for MD cases vs. AUD cases, measured from the second model. A value higher than 1 would indicate that AUD is more specific in risk transmission in comparison to MD. Variability estimates are retrieved from using non-parametrical bootstrapping techniques, along with the percentile method and 2000 bootstrap samples.

For testing whether the transmission of risk for AUD is more specific in comparison to MD, we include all offspring cases with AUD only (*n* = 18,345) or MD only (*n* = 174,011), defining the AUD only individuals as cases and MD only as controls. Logistic regression with case status as the dependent variable is performed, including all different dual mating combinations as independent variables, with the null × null combination as the reference level, and further controlled for birth years of offspring and parents, as well as the sex of offspring. We define *F* as the fraction with the nominator being the odds ratio (OR) for being a case, dependent on the parental dual diagnostic combination of one AUD diagnosis only, and with the denominator being the OR for being a control, dependent on the parental dual diagnostic combination of one MD diagnosis only. Variability estimates are retrieved from using non-parametrical bootstrapping techniques, along with the percentile method and 2000 bootstrap samples.

Levels of significance 0.05, 0.01, 0.001, and 0.0001 were marked in tables. Data analysis was conducted from October 4, 2024, to February 19, 2025. Statistical analyses were performed using R, version 4.4.2 (R Core Team 2024) (Table A3) and SAS, version 9.4 (SAS Institute 2012).

## 3 ∣ Results

Table 1 provides descriptive statistics of the sample of 1,244,516 offspring of intact mother–father parental pairs that we examine here. At the time of last follow-up, parents were in their mid-to-late 60s, so they were almost entirely through the age at risk for MD and AUD. Their children were, on average, in their late 30s. In the children's generation, who lived at a time when the registries were largely complete and functioning well, the mean age at onset of AUD was in the late 20s and, of MD, the early 30s. In parents who had completed their ages at risk for MD and AUD, the lifetime prevalence for MD in mothers (16.1%) was 70% higher than that seen in fathers (9.5%), while the risk for AUD in fathers (6.2%) was three times that in mothers (2.1%). The rates of MD were nearly twice as high in daughters (10.0%) as in sons (9.5%), while the rate of AUD was over twice as high in sons (3.8%) as in daughters (1.6%).

**FIGURE 1 ∣:**
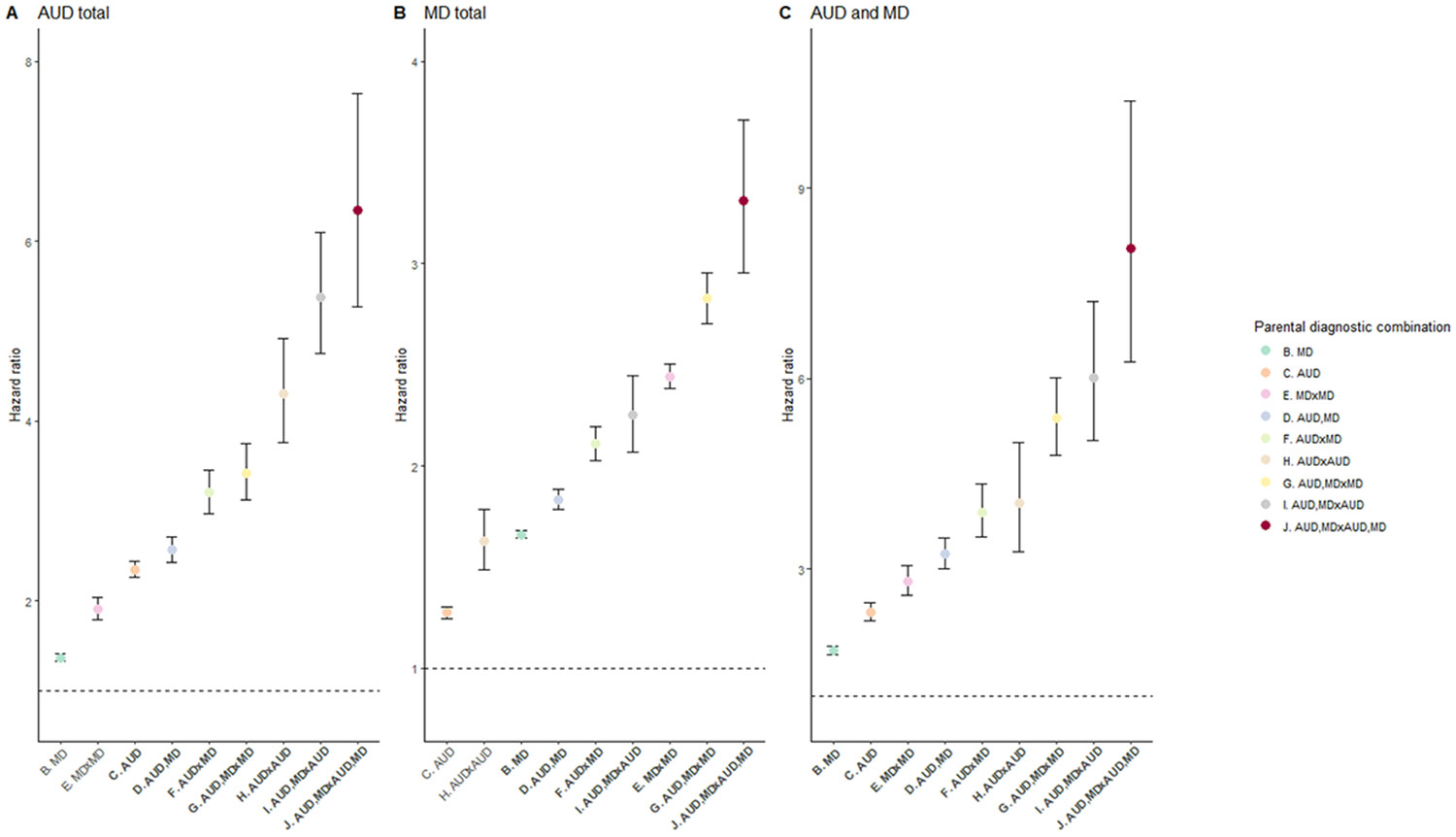
(A–C) The impact of mating combinations of parental alcohol use disorder (AUD) and major depression (MD) on the Hazard Ratio in Offspring for AUD and MD. (A) The hazard ratio (HR) and 95% CIs on the *y*-axis for the risk for AUD in offspring as a function of nine parental mating types listed to the right of the figure and keyed to Table 2. The mating types are given in order of their increasing HR for AUD. (B) The hazard ratio (HR) and 95% CIs on the *y*-axis for the risk for MD in offspring as a function of nine parental mating types listed to the right of the figure and keyed to Table 2. The mating types are given in order of their increasing HR for MD. (C) The hazard ratio (HR) and 95% CIs on the y-axis for the risk for comorbid MD^+^AUD in offspring as a function of 9 parental mating types listed to the right of the figure and keyed to Table 2. The mating types are given in order of their increasing HR for having both MD and AUD.

Table 2 describes the hazard ratios (HRs) and 95% CIs for the nine possible mating types accounting for three possible diagnoses in parents, AUD, MD, or both. These analyses ignored parental sex, and all were compared to a baseline model where neither parent had ever had a diagnosis of either MD or AUD. The best way to approach explaining the results in this table is to walk through the findings for our three outcomes—total AUD, total MD, and comorbid MD/AUD—one at a time. We will begin with “total AUD” as depicted in Figure 1a, and for simplicity, just quote the resulting HRs. The models we mention here are bolded in the figure, and *p*-values for all the model comparisons are presented in Table A4.

We start with Model C, that is, our AUD × unaffected (UN) parental mating type. Offspring from this mating have an HR for AUD of 2.35 compared to baseline mating type. The next step is Model D—an AUD/MD × UN mating type, which results in a modest further increased HR to 2.57. Next, we examine Model F—the AUD × MD mating type—where the HR jumps to 3.21. If the parent in Model F who has AUD also has MD (Model G—an AUD/MD × MD mating type), the HR increases slightly higher to 3.42. We then progress to Model H—an AUD × AUD mating type—and the HR grows to 4.30. From Model H, Models I and J make first one and then both parents comorbid with MD (i.e., respectively, an AUD/MD × AUD and an AUD/MD × AUD/MD mating type), and the HRs for offspring AUD go up to, respectively, 5.38 and 6.34.

Let's now illustrate our results for a pathway of models examining offspring risk for total MD (Figure 1b). We start with Model B, our MD × UN mating type, which produces an HR for MD in offspring of 1.66. If that same parent also has AUD (Model D—an MD/AUD × UN mating), the MD risk increases slightly to 1.84. Next is Model F—an MD × AUD mating—which produces an HD for MD that is modestly higher: 2.11. Going now to Model E—our MD × MD mating—the HR for MD increases to 2.44. This risk then increases modestly when one parent (Model G—an MD/AUD × MD mating) or both parents (Model J MD/AUD × MD/AUD) are also diagnosed with AUD: HR, respectively, 2.83 and 3.31.

The final analysis in Table 2 considers prediction of offspring AUD/MD comorbidity (Figure 1c). We begin by noting that Model C—our AUD × UN mating—contributes more strongly to comorbid offspring MD/AUD (HR = 2.33) than does an MD × UN mating (Model B, HR = 1.72). Model D—an AUD/MD × UN mating—produces a substantial risk for comorbid offspring (HR = 3.24) but not as strongly as (Model F—an AUD × MD mating: HR = 3.90). Surprisingly, Model H—our AUD × AUD mating—increased offspring comorbid risk slightly more (HR = 4.04). Rates of child comorbidity go up considerably with Model I—the AUD/MD × AUD mating (HR = 6.02) and take another jump if both parents are comorbid (Model J—an AUD/MD × AUD/MD mating: HR = 8.06).

Having described the results of our nine different mating types one by one, where we did not distinguish between mothers and fathers, we turn to Table 3, which addresses this question, examining the outcome in all the offspring together (along with significant differences across estimates in Table A5). Focusing initially on predicting total offspring AUD, the impact of mothers and fathers with AUD are similar and not significantly different (with HRs ~2.30), while mother's MD is a significantly stronger predictor of AUD (HR = 1.42) than is father's MD (HR = 1.29) (HR = 1.10 between parents, *p* < 0.0001). In predicting total MD, mother's MD (HR = 1.70) is significantly stronger than father's MD (HR = 1.58) (HR = 1.08 between parents, *p* < 0.0001), and the same is true for AUD (HRs of 1.33 vs. 1.25; HR between parents = 1.06, *p* < 0.05). In the prediction of childhood MD/AUD comorbidity, mother's MD (HR = 1.77) is also significantly stronger than father's MD (HR = 1.56) (between parent HR = 1.15, *p* < 0.0001), while no difference is seen for mother's vs. father's AUD (HRs = 2.17 vs. 2.29).

Table 3 then examines two-way interactions within and between parents. For five of six predictions for mother's AUD × mother's MD and father's AUD × father's MD, the interactions are below one. This means that the impact of a comorbid parent on offspring risk is less than the sum of the independent effects of having only MD and having only AUD. Of note, none of the four three-way interactions or the one four-way interaction between parental diagnoses were statistically significant.

Table 4 explores the main effects of the impact of parental AUD and MD on offspring risk for AUD and MD, taking into account parental and offspring sex. The impact of the mother's AUD on risk for AUD, MD, and comorbid MD/AUD does not differ in sons versus daughters. By contrast, MD in the mother has significantly stronger effects on risk in daughters than sons for AUD and comorbid MD/AUD, but not for MD. AUD in fathers impacts differently in his children only for MD risk, where it is significantly stronger in sons than daughters. Father's MD is significantly stronger in its impact risk in sons versus daughters for both MD and comorbid AUD/MD. We also explored the interactions of parental AUD and MD on offspring AUD and MD, but that is of more limited interest and is presented in Table A6.

Finally, we examine the question of the specificity of the parent-offspring transmission of MD and AUD. We find AUD transmission to be more specific in comparison to MD using the approach outlined in the methods section, where we specifically define the parameter *F* as the odds of being an AUD case vs. an MD case for dual combinations of either one AUD diagnosis or one MD diagnosis. An *F* value of unity would mean equal specificity of the transmission of the MD and AUD risk across generations. Our estimate of *F* with a 95% CI is 1.36 (1.26–1.47; *p* < 0.001), meaning the AUD parent-offspring transmission is more specific than that of MD.

## 4 ∣ Discussion

We sought, in this paper, to utilize a heterotypic dual mating design to explore the cross-generational transmission of two frequently comorbid disorders: MD and AUD. Our main goal was to use this design to gain further insight into the familial contribution to their comorbidity. Our samples of parents and children were large and therefore gave us considerable statistical power to examine subtle aspects of the patterns of transmission and co-transmission. We review the seven major findings in our analyses.

First, for both MD and AUD, we saw consistent patterns of direct parent–child transmission regardless of the background pattern of other parental disorders. That is, these results occurred on a background of no other parental disorders—UN × UN → UN × A—(where A is MD or AUD) or going from one parent affected to both parents affected (UN × A → A × A). This was also seen when the other parent had the other disorder (here B), so B × UN → B × A, as well as when the same parent had the other disorder (B × UN → A/B × UN), as well as with higher comorbid combinations.

Second, we saw consistent cross-transmission—that is, from parental AUD to offspring MD and parental MD to offspring AUD. This occurred without other parental psychopathology, as well as when the other parent had MD or AUD. This was true not only for total MD or total AUD, as seen in Table 1, but also for only MD (i.e., without AUD comorbidity) and only AUD (without MD comorbidity) (see Table A7). However, across many combinations of mating types, the cross-disorder cross-generational transmissions for MD → AUD and AUD → MD were consistently weaker than the within-disorder cross-generational transmissions: MD → MD and AUD → AUD. These results, consistent with most prior twin and family studies—but not all, see Maier et al. (1994)—showing a familial/genetic correlation between MD and AUD (Kendler et al. 1993; Nurnberger Jr. et al. 2004), provide robust evidence for a sharing of the familial liability across the two disorders.

Third, we could further test the hypothesis of a sharing of familial risk between MD and AUD by comparing the impact of offspring risk from both MD and AUD from the same parent. The interactions, as seen in Table 3, are negative and significant in both fathers and mothers. This is because of the sharing of familial risk between MD and AUD; the impact of risk to children of one disorder added to the other in the same parent is less than their independent impact.

Fourth, we could also examine a subtler question. Does the impact of parental illness on child risk vary as a function of the presence of the same disorder in the other parent? As seen in Table 3, the answer to that question was yes for both MD and AUD; the interaction was negative for both disorders. That is, while the risk to the offspring from having two parents with MD or AUD was substantially greater than from just one, the risk was less than that predicted for the sum of the individual effects taken on their own.

Fifth, we were specifically interested in assessing whether the comorbid MD/AUD syndrome was itself specifically transmitted across generations. This idea—that a comorbid combination of two disorders could actually constitute an independent disorder—was contained in the comorbidity typology of Klein and Riso (1993) and then formally statistically modeled by Neale and Kendler (1995). Our test for this hypothesis can be best seen by the comparison of Models D and F in Table 2. In Model D, one parent had comorbid MD/AUD, and the other parent had neither. In Model F, one parent had MD and the other AUD. If the cases of MD/AUD were truly an independent disorder, one would expect the risk for MD/AUD in offspring to be higher in Model D than Model F. By contrast, if the two disorders were independent but had a correlated liability, the total risk from the comorbid parent in Model D would actually be less than that received from the two independent disorders present in both parents in Model F. The results show that the HR of comorbid MD/AUD in Model D was 3.24 and, in Model E, was significantly higher (*p* = 0.004) at 3.90. These results are inconsistent with the idea that MD/AUD constitutes an independent diagnostic entity transmitted in families.

Sixth, we were also able to explore the impact of sex on parent-offspring transmission of AUD and MD. Comparing results from mothers versus from fathers (Table 3), we observed four significant differences for MD → AUD, MD → MD, AUD → MD, and AUD/MD → MD. In every case, maternal transmission to offspring was stronger than paternal transmission. Then, in Table 4, we examined the sex of both parents and offspring, where we had five significant differences. All of them demonstrated significantly stronger transmission in same-sex versus opposite-sex parent-offspring pairs: Mother's MD → AUD; Mother's MD → AUD/MD; Father's AUD → MD; Father's MD → MD; Father's MD → AUD/MD. Only the mother's AUD did not transmit differently in sons versus daughters. Four plausible mechanisms could be responsible for these differences, which we are not, unfortunately, well able to differentiate. First, intrauterine effects, which might be more plausible for AUD than MD, would predict stronger transmission from mothers to both sons and daughters (Riley et al. 2011; Yates et al. 1998). Non-paternity estimates, which are generally low in European samples (Ryman and Chakraborty 1982; Sasse et al. 1994; Wolf et al. 2012), would also predict increased maternal vs. paternal effects for all syndromes. Psychological effects, a broad term that could encompass many specific mechanisms, are typically seen as predicting a stronger overall maternal effect but might also predict same-sex transmission to be stronger than opposite-sex. Finally, risk genes on the X-chromosome predict stronger mother-to-son versus father-to-son transmission. Our prediction of stronger father-to-son transmission than father-to-daughter transmission for MD → MD and AUD → MD is not consistent with a substantial non-paternity rate or X-chromosomal effects. We also find no evidence of a stronger parent-offspring transmission of AUD from mothers versus fathers, which has been predicted from increased in utero exposure to higher alcohol levels in mothers with heavy drinking (Yates et al. 1998). We were, interestingly, unable to replicate prior evidence of an absence of cross-sex genetic correlation between MD and AUD (Prescott et al. 2000), although we did show evidence for stronger within cross-sex parent-offspring transmission for both disorders.

Seventh, we were in a position to compare rigorously the specificity of transmission of our two disorders. As a review of the HRs in Table 2 would suggest, the familial transmission of MD is less specific than that of AUD. This is consistent with prior evidence from an analysis of family genetic risk scores in Sweden that showed genetic risk to be among the least diagnostic specific of any genetic risk scores for major psychiatric and substance use disorders (Kendler et al. 2021).

Because our study was conducted in intact families, we are not able to determine directly the degree to which the pattern of findings results from genetic versus shared environmental effects. While the major meta-analysis of twin studies of MD (Sullivan et al. 2000) and AUD (Verhulst et al. 2015) suggests that shared environmental effects make no significant contribution to family resemblance for MD and only a modest impact on AUD, our expanded adoption study of MD suggests that a substantial proportion of the parent-offspring transmission of MD might result from familial-environmental factors (Kendler, Ohlsson, et al. 2018). While we would suspect that a large proportion of the effects seen in this report results from the impact of the transmission of genetic effects from parents to their offspring, it is also plausible that familial-environmental factors are playing an important role.

It is also of interest to consider how our findings, which focus on the clinical syndromes, might relate to what is occurring at the level of genetic risk. We previously examined how the level of genetic risk for MD and AUD impacted disease risk and generally found evidence for positive interactions (using additive models) (Kendler et al. 2022). These findings are broadly consistent with our results obtained here using a quite different method—the dual mating strategy. This can be seen most clearly in Table 2 where, for example, the risk for AUD is considerably higher in the offspring of matings with one parent with AUD and the other with MD versus only one parent with AUD. Parallel results are seen for MD.

### 4.1 ∣ Limitations

These results should be considered in the context of three potentially important methodological limitations. First, the quality of these analyses depends substantially on the validity of the Swedish registry diagnoses of AUD and MD. For AUD, we utilized contact, both with the medical and criminal registries, and, as suggested by our prevalence estimates, are studying a more severely affected group than would be detected at interview using the current wide DSM-5 AUD disorder (American Psychiatric Association 2013; Grant et al. 2015). The validity of the AUD diagnosis is supported both by the high rates of concordance across ascertainment methods (Kendler et al. 2015) and the patterns of resemblance in relatives similar to those found in personally interviewed samples (Prescott and Kendler 1999). For MD, we relied only on diagnoses obtained from medical contacts, and we know that a large proportion of our cases were only seen in primary care (Kendler, Ohlsson, Lichtenstein, et al. 2018). The validity of our MD diagnoses is supported by evidence that its prevalence, sex ratio, sibling, and twin correlations, and associations with psychosocial risk factors (Kendler, Ohlsson, Lichtenstein, et al. 2018; Sundquist et al. 2017) are consistent with many prior studies of the syndrome.

Second, we did not formally correct for spousal correlations for MD and AUD. We present the relevant correlations in Table A8, and they are modest to moderate as expressed by tetrachoric correlations: +0.30 for AUD and +0.19 for MD. Our figure for MD is similar to that reported previously, while our marital correlation for AUD is somewhat higher (Maes et al. 1998). The cross-correlations were especially modest: +0.10 for maternal MD and paternal AUD, and +0.09 for maternal AUD and paternal MD. If the majority of these correlations are due to assortative mating, rather than spousal interaction (although we have strong evidence for a causal effect of spousal AUD on individual AUD risk (Kendler, Lonn, Salvatore, et al. 2018)), and have been stable over generations, then this process could contribute to the shared familial risk for AUD and MD (Border et al. 2022; Maes et al. 1998).

Third, we also had information about the respective ages of first contact for AUD and MD in the parents in our sample who had both diagnoses. Interestingly, none of the tests we applied to see if risk to offspring differed by order of diagnoses were even nominally significant. So, this was one potentially complicating feature of our design with which we did not need to be concerned.

## 5 ∣ Conclusions

What have we learned in these extensive analyses about the familial nature of the comorbidity between MD and AUD? Most importantly, we showed strong evidence in favor of a shared familial liability that is transmitted symmetrically across generations, meaning that MD in a parent predisposed to AUD in the offspring and vice-versa. But we also showed that the cross-disorder cross-generational transmission was consistently weaker than the within-disorder cross-generational effects, which would be expected if there was not complete overlap in the familial liability to the two disorders. We could reject the hypothesis that a combined AUD/MD syndrome was itself specifically transmitted across generations. Subtle sex effects were seen, showing that maternal-offspring transmissions were modestly stronger in most comparisons than paternal-offspring, as were same-sex versus opposite-sex parent-offspring effects. The heterotypic dual-mating strategy holds promise as a methodology for clarifying features of the relationship between the familial transmission of pairs of potentially related disorders.

## Figures and Tables

**TABLE 1 ∣:** Descriptive statistics for sample size, birth year, age, sex distributions, parental and offspring prevalences and ages at first registration of alcohol use disorder (AUD) and major depression (MD).

	Offspring						Biological parent			
	All		Female		Male		Mother		Father	
Number	1,244,516		598,701		645,815		1,244,516		1,244,516	
	Mean	SD	Mean	SD	Mean	SD	Mean	SD	Mean	SD
Year of birth	1979.9	6.1	1979.9	6.1	1979.8	6.1	1951.7	7.1	1949.0	7.5
Age at follow-up	37.7	6.8	37.5	6.9	37.8	6.8	66.2	7.2	68.3	7.3
Prevalence rates (%)										
AUD total	2.72		1.59		3.77		2.12		6.15	
MD total	15.23		20.00		10.80		16.08		9.50	
AUD & MD	1.25		1.00		1.48		1.01		1.75	
Age at first diagnosis										
	Mean	SD	Mean	SD	Mean	SD	Mean	SD	Mean	SD
AUD	28.3	8.2	28.7	8.6	28.1	8.1	55.2	10.6	52.3	14.6
MD	31.0	7.0	30.7	7.0	31.4	7.0	56.0	10.4	59.3	10.9

**TABLE 2 ∣:** The Impact of the Nine Possible Mating Combinations of Parental Alcohol Use Disorder (AUD) and Major Depression (MD) on the Hazard Ratio (HR) in Offspring for total AUD and total MD. “Null” means a parent without a diagnosis of MD or AUD. A superscript of + means affected and of − means unaffected.

	A. null × null	B. AUD^−^MD ^+^ × null		C. AUD^+^MD^−^ × null		D. AUD^+^MD^+^ × null		E. AUD^−^MD^+^ × AUD^−^MD^+^		F. AUD^−^MD^+^ × AUD^+^MD^−^		G. AUD^+^MD^+^ × AUD^−^MD^+^		H. AUD^+^MD^−^ × AUD^+^MD^−^		I. AUD^+^MD^+^ × AUD^+^MD^−^		J. AUD^+^MD^+^ × AUD^+^MD^+^	
Number of offspring	901,933	221,729		52,262		24,313		23,098		9668		6329		2240		2105		839	
Offspring diagnosis		HR	95% CI	HR	95% CI	HR	95% CI	HR	95% CI	HR	95% CI	HR	95% CI	HR	95% CI	HR	95% CI	HR	95% CI
AUD total	Ref	1.37	1.34, 1.41****	2.35	2.26, 2.45****	2.57	2.43, 2.71****	1.91	1.79, 2.03****	3.21	2.97, 3.46****	3.42	3.13, 3.75****	4.30	3.76, 4.92****	5.38	4.75, 6.09****	6.34	5.27, 7.64****
MD total	Ref	1.66	1.64, 1.68****	1.28	1.25, 1.30****	1.84	1.79, 1.39****	2.44	2.38, 2.50****	2.11	2.03, 2.20****	2.83	2.70, 2.96****	1.63	1.49, 1.79****	2.25	2.07, 2.45****	3.31	2.95, 3.71****
AUD & MD	Ref	1.72	1.65, 1.79****	2.33	2.19, 2.47****	3.24	3.00, 3.49****	2.81	2.59, 3.05****	3.90	3.50, 4.34****	5.37	4.80, 6.02****	4.04	3.27, 5.00****	6.02	5.02, 7.21****	8.06	6.27, 10.37****

*Note:* Significance levels for p-values:

* < 0.05

** < 0.01

*** < 0.001

**** < 0.0001.

**TABLE 3 ∣:** The impact of mating combinations of parental alcohol use disorder (AUD) and major depression (MD) on the hazard ratio (HR) in offspring for AUD and MD—sex-specific parental effects model based on significant parental main and interaction effects.

Independent variables of parental diagnoses	Offspring diagnosis					
	AUD total		MD total		Comorbid AUD & MD	
	HR	95% CI	HR	95% CI	HR	95% CI
Mother's AUD	2.30	2.13, 2.48****	1.33	1.27, 1.38****	2.17	2.00, 2.35****
Mother's MD	1.42	1.39, 1.46****	1.70	1.68, 1.71****	1.77	1.71, 1.84****
Father's AUD	2.32	2.24, 2.41****	1.25	1.22, 1.28****	2.29	2.17, 2.43****
Father's MD	1.29	1.24, 1.34****	1.58	1.55, 1.60****	1.56	1.48, 1.64****
Mother's AUD × Mother's MD	0.83	0.75, 0.91***	0.89	0.84, 0.94****	^a^	
Mother's AUD × Father's AUD	0.86	0.77, 0.95**	^a^		0.75	0.65, 0.87***
Mother's AUD × Father's MD	^a^		^a^		^a^	
Mother's MD × Father's AUD	^a^		^a^		^a^	
Mother's MD × Father's MD	^a^		0.92	0.90, 0.95****	^a^	
Father's AUD × Father's MD	0.83	0.77, 0.90****	0.89	0.86, 0.92***	0.82	0.74, 0.91***
Mother's AUD × Mother's MD × Father's AUD	^a^		^a^		^a^	
Mother's AUD × Mother's MD × Father's MD	^a^		^a^		^a^	
Mother's AUD × Father's AUD × Father's MD	^a^		^a^		^a^	
Mother's MD × Father's AUD × Father's MD	^a^		^a^		^a^	
Mother's AUD × Mother's MD × Father's AUD × Father's	^a^		^a^		^a^	

*Note:* Significance levels for *p*-values:

* < 0.05

** < 0.01

*** < 0.001

**** < 0.0001.

a Not significant at significance level *p* < 0.05.

**TABLE 4 ∣:** The impact of mating combinations of parental alcohol use disorder (AUD) and major depression (MD) on the hazard ratio (HR) in offspring for AUD and MD—sex-specific parental with sex-specific offspring effects model based on significant main effects.

Independent variables of parental diagnoses		Offspring diagnosis						
		Offspring sex	AUD total		MD total		Comorbid AUD & MD	
			HR	95% CI	HR	95% CI	HR	95% CI
Main effects	Mother's AUD	Male	2.36	2.17, 2.56****	1.32	1.27, 1.38****	2.17	2.00, 2.35****
		Female	^a^		^a^		^a^	
		*p*						
	Mother's MD	Male	1.37	1.32, 1.41****	1.70	1.68, 1.72****	1.72	1.64, 1.80****
		Female	1.58	1.50, 1.66****	^a^		1.86	1.76, 1.97****
		*p*		< 0.0001				0.0289
	Father's AUD	Male	2.33	2.24, 2.42****	1.30	1.26, 1.35****	2.30	2.17, 2.43****
		Female	^a^		1.22	1.19, 1.25****	^a^	
		*p*				0.0012		
	Father's MD	Male	1.29	1.24, 1.34****	1.66	1.62, 1.70****	1.64	1.54, 1.74****
		Female	^a^		1.53	1.50, 1.56****	1.44	1.37, 1.56****
		*p*				< 0.0001		0.0056

*Note:* Significant interaction effects were included in the model fittings, and are shown in the Table A6. Significance levels for *p*-values:

* < 0.05

** < 0.01

*** < 0.001

**** < 0.0001.

a Sex-specific effect for female not shown if not significantly different to male effect at significance level *p* < 0.05.

## Data Availability

The data for this study are not publicly available due to legal restrictions with regard to the nationwide Swedish registers, but they can be acquired directly from the responsible authorities pending their approval.

## Appendix A

**TABLE A1 ∣:** Description of registers.

Total Population Register
The Total Population Register was created digitally in 1968 and includes yearly information, from the National Tax Board, on individuals registered in Sweden. It is possible to retrieve information on, for example, birth, death, immigration, emigration, migration within Sweden, place of residence, family information, civil status, etc. For more information, see https://www.scb.se/contentassets/8f66bcf5abc34d0b98afa4fcbfc0e060/rtb-bar-2016-eng.pdf
Multi-Generation Register
The Multi-Generation Register is a register made up of persons who have been registered in Sweden at some time since 1961 and those who were born in 1932 or later. These are called index persons. The register contains connections between index persons and their biological parents. In 2024, more than 11 million index persons were included in the register. The Multi-Generation Register is a part of the register system for Total Population Register, where information comes from the National Tax Board. Every year, a new version of the register is created, including new index persons who immigrated or were born during the year. Information from the Multi-Generation Register may be disclosed for research and statistical purposes. See https://www.scb.se/vara-tjanster/bestall-data-och-statistik/register/flergenerationsregistret/
National Patient Register
In the 1960's the National Board of Health and Welfare started to collect information regarding in-patients at public hospitals, the National Patient Register (NPR). Initially it contained information about all patients treated in psychiatric care and approximately 16% of patients in somatic care. The register at that time covered six of the 26 county councils in Sweden. In 1984, the Ministry of Health and Welfare together with the Federation of County Councils decided a mandatory participation for all county councils. From 1987, NPR includes all in-patient care in Sweden. Since 2001, the register also covers outpatient doctor visits including day surgery and psychiatric care from both private and public caregivers. For more information, see https://www.socialstyrelsen.se/en/statistics-and-data/registers/national-patient-register/
Primary Care Data
We also used information from our new Primary Care research dataset including individual-level information on clinical diagnoses from primary health care centers from the following Swedish counties: Blekinge (2009–2018), Dalarna (2005–2018), Gotland (2011–2018), Gävleborg (2010–2018), Halland (2007–2018), Jönköping (2008–2018), Kalmar (2007–2018), Kronoberg (2006–2018), Norrbotten (2001–2018), Skåne (1989–2018), Stockholm (2003–2018), Södermanland (1992–2018), Uppsala (2005–2018), Västra Götaland (2000–2018), Värmland (2005–2018), Västerbotten (1991–2018), Västernorrland (2008–2018), Västmanland (2014–2018), Östergötland (1990–2018), and Örebro (2006–2018). The retrieval of data differs due to timing of digitalization of patient records. In 2018, 99% of the Swedish population lived in these 20 counties. For more information see Sundquist et al. (2017) common adult psychiatric disorders in Swedish primary care where most mental health patients are treated
The Population and Housing Censuses
Every fifth year between 1960 and 1990 Sweden conducted censuses. These registers include among other things, the population's employment, the composition of households and housing. For more information, see https://www.scb.se/en/finding-statistics/statistics-by-subject-area/population-and-living-conditions/population-composition-and-development/population-and-housing-census-1960-1990-tpr/
Prescribed Drug Register
The Swedish Prescribed Drug Register started in July 2005 and includes all prescribed drugs being fetched at pharmacies, linked to personal numbers. See https://www.socialstyrelsen.se/en/statistics-and-data/registers/national-prescribed-drug-register/
Cause of Death Register
The Cause of Death Register includes all deaths occurring in Sweden from 1961 (including for Swedish citizens dying abroad) and is updated yearly. There is also a historical register between the years 1952 to 1960. For more information, see https://www.socialstyrelsen.se/en/statistics-and-data/registers/national-cause-of-death-egister/
Criminal and Suspicion Register
The Swedish Criminal Register and the Swedish Suspicion Register includes individual-level information on all committed crimes from 1973 and all suspicions of crimes related to an individual from 1998. For more information, see https://polisen.se/lagar-och-regler/behandling-av-personuppgifter/polisens-register/

**TABLE A2 ∣:** Definition of alcohol use disorder and major depression.

	Registers used	Definition
Alcohol Use disorder (AUD)	Hospital Discharge Register; Outpatient Care Register; Primary Care Data; Prescribed Drug Register; Cause of Death Register; Criminal Register; Suspicion Register	Alcohol Use Disorder (AUD) was identified in the Swedish medical and mortality registries by ICD codes: ICD8: 571.0, 291, 303; ICD9: V79B, 305A, 357F, 571A-D, 425F, 535D, 291, 303; ICD 10: E24.4, G31.2, G62.1, G72.1, I42.6, K29.2, K70, K85.2, K86.0, O35.4, F10.1-F10.9; in the Swedish Criminal Register and the Swedish Suspicion Register with at least two registrations of drunk driving (suspicion code 3005, law 1951:649 (paragraph 4 and 4A)) or drunk in charge of a maritime vessel (suspicion code 3201, law 1994:1009 (chapter 20, paragraph 4 and 5)); in the Prescribed Drug Register by the drugs disulfiram (Anatomical Therapeutic Chemical (ATC) Classification System N07BB01), acamprosate (N07BB03), and naltrexone (N07BB04)
Major depression (MD)	Hospital Discharge Register; Outpatient Care Register; Primary Care Data	Major Depression (MD) was identified in the Swedish medical registries by ICD codes: ICD8: 296.0, 296.2, 298.0, 300.4; ICD9: 296B, 298A, 300E; ICD10: F32, F33

**TABLE A3 ∣:** Details on R-packages used in statistical analyses.

1. Therneau (2024). survival: A package for Survival Analysis in R. R package.
2. Wickham (2016). ggplot2: Elegant Graphics for Data Analysis. New York, NY: Springer-Verlag.
3. Wickham et al. (2023). haven: Import and Export “SPSS”, “Stata” and “SAS” Files. R package.
4. Wickham et al. (2023). dplyr: A Grammar of Data Manipulation. R package.
5. Barrett et al.(2024). Data. table: Extension of “data. frame”. R package.
6. Neuwirth (2022). RColorBrewer: ColorBrewer Palettes. R package.
7. Kassambara (2023). ggpubr: “ggplot2” Based Publication Ready Plots. R package
8. Wickham (2023). stringr: Simple, Consistent Wrappers for Common String Operations. R package

**TABLE A4 ∣:** Contrast p-values testing for differences between HR depending on mating type (180 Tests, Model 1).

	B.AUD^−^MD^+^ × null	C. AUD^+^MD^−^ × null	D. AUD^+^MD^+^ × null	E.AUD^−^MD^+^ × AUD^'^MD^+^	F.AUD^−^MD^+^ × AUD^+^MD^−^	G. AUD^+^MD^+^ × AUD^−^MD^+^	H. AUD^+^MD^−^ × AUD^+^MD^−^	I. AUD^+^MD^+^ × AUD^+^MD^−^	J. AUD^+^MD^+^ × AUD^+^MD^+^
B. AUD^−^MD^+^ × null	NA	< 0.0001*	< 0.0001*	< 0.0001*	< 0.0001*	< 0.0001*	< 0.0001*	< 0.0001*	< 0.0001*
C. AUD^+^MD^−^ × null	< 0.0001*; < 0.0001*	NA	< 0.0001*	< 0.0001*	< 0.0001*	0.0002*	< 0.0001*	< 0.0001*	< 0.0001*
D. AUD^+^MD^+^ × null	< 0.0001*; < 0.0001*	< 0.0001*; < 0.0001*	NA	< 0.0001*	0.004	0.001	0.05	< 0.0001*	< 0.0001*
E. AUD^−^MD^+^ × AUD^−^MD^+^	< 0.0001*; < 0.0001*	< 0.0001*; < 0.0001*	< 0.0001*; < 0.0001*	NA	< 0.0001*	< 0.0001*	0.002	0.29	0.004
F. AUD^−^MD^+^ × AUD^+^MD^−^	< 0.0001*; < 0.0001*	< 0.0001*; < 0.0001*	< 0.0001*; < 0.0001*	< 0.0001*; < 0.0001*	NA	< 0.0001*	0.77	< 0.0001*	< 0.0001*
G. AUD^+^MD^+^ × AUD^−^MD^+^	< 0.0001*; < 0.0001*	< 0.0001*; < 0.0001*	< 0.0001*; < 0.0001*	< 0.0001*; < 0.0001*	0.27; < 0.0001*	NA	0.02	< 0.0001*	< 0.0001*
H. AUD^+^MD^−^ × AUD^+^MD^−^	< 0.0001*; 0.68	< 0.0001*; < 0.0001*	< 0.0001*; 0.02	< 0.0001*; < 0.0001*	0.0002*; < 0.0001*	0.006; < 0.0001*	NA	0.005	< 0.0001*
I. AUD^+^MD^+^ × AUD^+^MD^−^	< 0.0001*; < 0.0001*	< 0.0001*; < 0.0001*	< 0.0001*; < 0.0001*	< 0.0001*; 0.07	< 0.0001*; 0.17	< 0.0001*; < 0.0001*	0.02; < 0.0001*	NA	0.06
J. AUD^+^MD^+^ × AUD^+^MD^+^	< 0.0001*; < 0.0001*	< 0.0001*; < 0.0001*	< 0.0001*; < 0.0001*	< 0.0001*; < 0.0001*	< 0.0001*; < 0.0001*	< 0.0001*; 0.01	0.0009; < 0.0001*	0.15; < 0.0001*	NA

*Note:* Top right: AUD & MD; bottom left: AUD Total; MD Total. AUD = alcohol use disorder, MD = major depression.

* Significant at Bonferroni-corrected levels of *p* < 0.05/180 = 0.00028.

**TABLE A5 ∣:** Combinations of parental alcohol use Disorder (AUD) and major depression (MD) on the hazard ratio (HR) in offspring for AUD and MD: tests of difference of sex-specific parental effects model based on Table 3.

Independent variables of parental diagnoses	Offspring diagnosis					
	AUD total		MD total		Comorbid AUD & MD^a^	
	HR	95% CI	HR	95% CI	HR	95% CI
Mother's AUD vs. Father's AUD	0.99	0.91, 1.08	1.06	1.01, 1.11*	1.01	0.89, 1.15
Mother's MD vs. Father's MD	1.10	1.05, 1.16****	1.08	1.06, 1.10****	1.15	1.07, 1.23****
Comorbid Mother vs. Comorbid Father (Main + Interaction Effects)	1.09	0.99, 1.19	1.14	1.09, 1.20****	1.25	1.11, 1.42***

*Note:* HR > 1.0 indicated stronger maternal vs. paternal transmission. Significance levels for *p*-values:

* < 0.05

** < 0.01

*** < 0.001

**** < 0.0001.

a Based on model including the insignificant effect of Mother's AUD × Mother's MD for reasons of comparability.

**TABLE A6 ∣:** The impact of mating combinations of parental alcohol use disorder (AUD) and major depression (MD) on the hazard ratio (HR) in offspring for AUD and MD—sex-specific parental with sex-specific offspring effects model based on significant interaction effects, fitted within the models presented in Table 4.

Independent variables of parental diagnoses		Offspring diagnosis					
Interaction effects^c^	Offspring sex	AUD total		AUD total		AUD total	
		HR	95% CI	HR	95% CI	HR	95% CI
Mother's AUD × Mother's MD	Male	0.79	0.70, 0.89****	0.89	0.84, 0.94****	^a^	
	Female	^b^		^b^		^b^	
	*p*						
Mother's AUD × Father's AUD	Male	0.79	0.69, 0.92**	^a^		0.84	0.71, 0.99*
	Female	^b^		^b^		0.61	0.48, 0.77****
	*p*						0.0189
Mother's AUD × Father's MD	Male	^a^		^a^		^a^	
	Female	^b^		^b^		^b^	
	*p*						
Mother's MD × Father's AUD	Male	^a^		^a^		^a^	
	Female	^b^		^b^		^b^	
	*p*						
Mother's MD × Father's MD	Male	^a^		0.92	0.89, 0.95****	^a^	
	Female	^b^		^b^		^b^	
	*p*						
Father's AUD × Father's MD	Male	0.83	0.77, 0.89****	0.84	0.79, 0.89****	0.82	0.74, 0.91***
	Female	^b^		0.92	0.88, 0.96***	^b^	
	*p*				0.0214		
Mother's AUD × Mother's MD × Father's AUD	Male	1.29	1.05, 1.59*	^a^		^a^	
	Female	0.86	0.64, 1.15	^b^		^b^	
	*p*		0.0040				

*Note:* Significance levels for *p*-values:

* < 0.05

** < 0.01

*** < 0.001

**** < 0.0001.

a Not significant at significance level *p* < 0.05.

b Sex-specific effect for female not shown if not significantly different to male effect at *p* < 0.05.

c The unpresented three- and four-way interaction effects were not significant.

**TABLE A7 ∣:** The Impact of the Nine Possible Mating Combinations of Parental Alcohol Use Disorder (AUD) and Major Depression (MD) on the Hazard Ratio (HR) in Offspring for AUD only, AUD total, MD only and MD total. “Null” means a parent without a diagnosis of MD or AUD. A superscript of + means affected and of − means unaffected.

Offspring diagnosis	Mating combinations																	
	B. AUD^−^MD^+^ × Null		C. AUD^+^MD^−^ × Null		D. AUD^+^MD^+^ × Null		E. AUD^−^MD^+^ × AUD^−^MD^+^		F. AUD^−^MD^+^ × AUD^+^MD^−^		G. AUD^+^MD^+^ × AUD^−^MD^+^		H. AUD^+^MD^−^ × AUD^+^MD^−^		I. AUD^+^MD^+^ × AUD^+^MD^−^		J. AUD^+^MD^+^ × AUD^+^MD^+^	
	HR	95% CI	HR	95% CI	HR	95% CI	HR	95% CI	HR	95% CI	HR	95% CI	HR	95% CI	HR	95% CI	HR	95% CI
AUD only	1.12	1.07, 1.16****	2.33	2.22, 2.46****	2.04	1.89, 2.21****	1.23	1.11, 1.37****	2.64	2.37, 2.94****	1.94	1.66, 2.27****	4.34	3.65, 5.15****	4.64	3.91, 5.51****	4.79	3.64, 6.31****
AUD total	1.37	1.34, 1.41****	2.35	2.26, 2.45****	2.57	2.43, 2.71****	1.91	1.79, 2.03****	3.21	2.97, 3.46****	3.42	3.13, 3.75****	4.30	3.76, 4.92****	5.38	4.75, 6.09****	6.34	5.27, 7.64****
MD only	1.64	1.62, 1.66****	1.18	1.15, 1.21****	1.69	1.64, 1.74****	2.36	2.30, 2.42****	1.91	1.83, 1.99****	2.49	2.38, 2.62****	1.40	1.26, 1.55****	1.86	1.69, 2.05****	2.69	2.37, 3.06****
MD total	1.66	1.64, 1.68****	1.28	1.25, 1.30****	1.84	1.79, 1.89****	2.44	2.38, 2.50****	2.11	2.03, 2.20****	2.83	2.70, 2.96****	1.63	1.49, 1.79****	2.25	2.07, 2.45****	3.31	2.95, 3.71****
AUD & MD	1.72	1.65, 1.79****	2.33	2.19, 2.47****	3.24	3.00, 3.49****	2.81	2.59, 3.05****	3.90	3.50, 4.34****	5.37	4.80, 6.02****	4.04	3.27, 5.00****	6.02	5.02, 7.21****	8.06	6.27, 10.37****

*Note:* Significance levels for p-values:

* < 0.05

** < 0.01

*** < 0.001

**** < 0.0001.

**TABLE A8 ∣:** Observed parental tetrachoric correlations along with 95% CIs between major depression (MD) and alcohol use disorder (AUD) in parents.

Paternal diagnoses	Maternal diagnoses			
	Null	AUD total	MD total	AUD & MD
Null	0.19 (0.19–0.19)****	−0.22 (−0.22–(−)0.21)****	−0.17 (−0.17–(−)0.17)****	−0.20 (−0.20–(−)0.20)****
AUD total	−0.14 (−0.14–(−)0.14)****	0.30 (0.30–0.30)****	0.10 (0.09–0.10)****	0.26 (0.25–0.26)****
MD total	−0.19 (−0.19–(−)0.19)****	0.09 (0.09–0.10)****	0.19 (0.19–0.20)****	0.14 (0.13–0.14)****
AUD & MD	−0.17 (−0.17–(−)0.17)****	0.22 (0.22–0.23)****	0.16 (0.15–0.16)****	0.24 (0.24–0.24)****

*Note:* Significance levels for *p*-values:

* < 0.05

** < 0.01

*** < 0.001

**** < 0.0001.
